# Supplementary figures and images for: Methods of tagSNP selection and other variables affecting imputation accuracy in swine
Source: BMC Genet. 2013 Feb 21;14:8. doi: 10.1186/1471-2156-14-8 (PMC3734000; doi:10.1186/1471-2156-14-8)

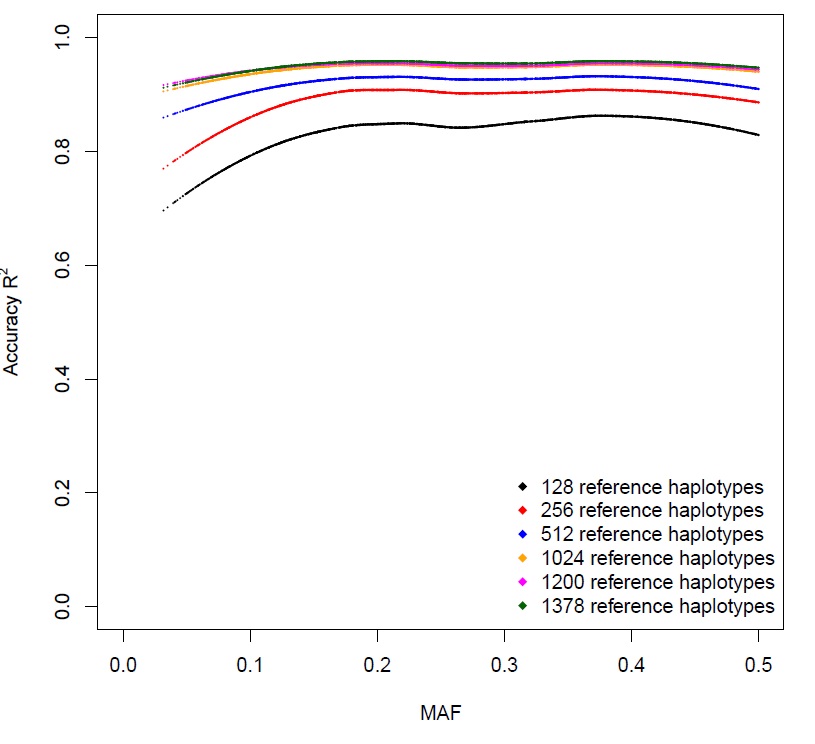

Supplement: Additional file 1: Figure S1 — Effect of reference panel size on imputation accuracy of SNP as a function of their MAF. Weighted mean average imputation accuracy (quantified as r2) as a function of MAF depicted for imputation based on haplotype reference panels of increasing size. [file 1471-2156-14-8-S1.jpg]

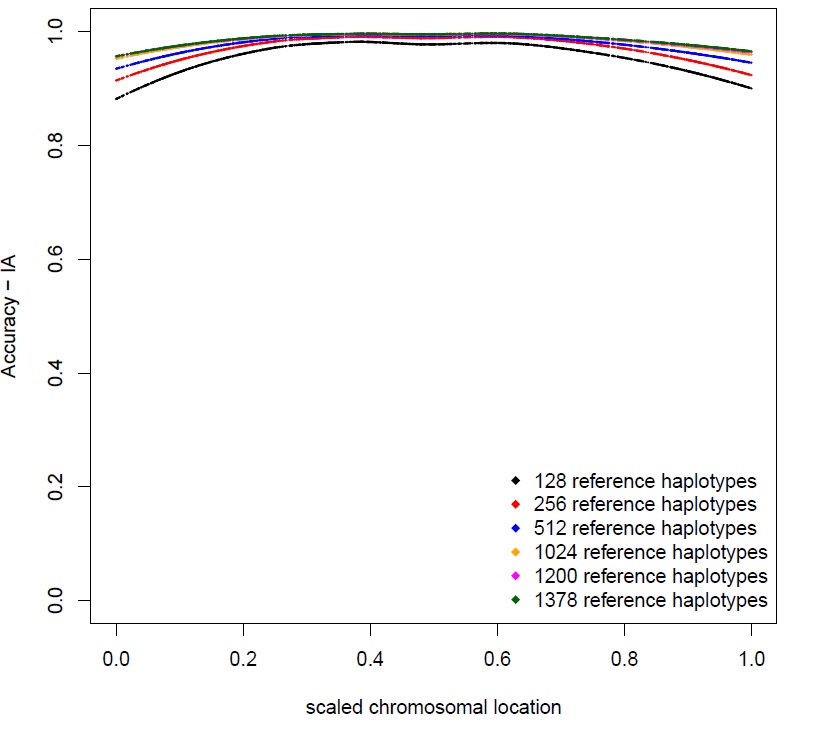

Supplement: Additional file 2: Figure S2 — Effect of reference panel size on imputation accuracy of SNP as a function of scaled physical location. Weighted mean average imputation accuracy (quantified as IA) as a function of the scaled chromosomal location for imputation based on haplotype reference panels of increasing size. [file 1471-2156-14-8-S2.jpg]
